# Designing a Compact Filtering Quasi-Yagi Antenna with Multiple Radiation Nulls Using Embedded Resistor-Loaded Arms

**DOI:** 10.3390/mi14071445

**Published:** 2023-07-19

**Authors:** Lipeng Zhai, Yi Guo, Zongming Xu, Xuefeng Zhang, Yanyun Chen, Jin Shi

**Affiliations:** 1 School of Information Science and Technology, Nantong University, Nantong 226019, China; zlp2952893072@outlook.com (L.Z.); gy1458708657@outlook.com (Y.G.); xefeng@hotmail.com (X.Z.); chenyy_ntu@outlook.com (Y.C.); 2 Zhongtian Radio Frequency Cable Co., Ltd., Nantong 226010, China; xuzm@chinaztt.com; 3 Research Center for Intelligent Information Technology, Nantong University, Nantong 226019, China; 4 Nantong Key Laboratory of Advanced Microwave Technology, Nantong University, Nantong 226019, China

**Keywords:** compact, embedded, filtering antenna, quasi-Yagi antenna, multiple radiation nulls

## Abstract

In this paper, a compact quasi-Yagi antenna with embedded resistor-loaded arms is proposed to obtain a filtering response with four radiation nulls. The embedded resistor-loaded arms achieve two additional radiation nulls caused by reverse currents and absorb the unwanted out-of-band resonant points brought by themselves. The director close to the driver provides a resonant point and a radiation null caused by opposite currents between the driver and the director. Compared with other filtering quasi-Yagi antennas, the proposed one can achieve a filtering response with a compact size along the endfire direction. For demonstration, a balun-integrated prototype covering the 5G band N78 (3.3–3.8 GHz) is designed with the size along the endfire direction (without ground) of 0.13 λ_0_ (λ_0_ is the wavelength in the free space at center frequency), and the measured results show a 10 dB impedance-matching bandwidth of 22.9% (3.21–4.04 GHz), four radiation nulls, and a peak gain of 4.73 dBi.

## 1. Introduction

The quasi-Yagi antenna, as an important planar endfire antenna, has attracted great attention owing to its advantages of having a planar structure and its ease of fabrication and installation [1,2]. The filtering quasi-Yagi antenna integrating the filtering function and endfire radiation can further reduce the size and loss of the system and enhance the integration level of the system [3]. Although the filtering quasi-Yagi antenna integrates two functions into one component, whether the compact size is really achieved in the filtering quasi-Yagi antenna is one of the important issues.

Some methods for designing a filtering quasi-Yagi antenna were reported. One method is cascading the filtering structure before the quasi-Yagi antenna, where the filtering structures, such as the bandpass filtering structure [4,5,6,7,8,9,10,11], the bandstop filtering structure [12], and the filtering balun [13,14,15,16,17], determine the filtering response. Nevertheless, the filtering structures increase the whole size, especially in the endfire direction, and additional loss is inevitable. Adding the parasitic structure into the quasi-Yagi antenna is another method for creating a filtering quasi-Yagi antenna, for example, inserting a double-sided parallel-strip line (DSPSL) filter between the driver and the reflector to enhance the frequency selectivity [18], which adds a parasitic structure between the driver and director to produce two radiation nulls [19,20]. Although the size of the antenna is not increased when introducing the filtering response, the size along the endfire direction is still large to maintain the performance.

On the other hand, different kinds of resistor-loaded structures have been utilized in antennas to solve different problems. The first kind consists of adding a combination of one stub and one resistor-loaded stub before an antipodal tapered slot antenna to achieve a reflectionless notch response in the wideband antenna [21]. The second kind consists of loading a piece of graphene nanoplate as a tunable resistor to the feed line to obtain the gain manipulation function [22]. The third kind consist of adding a resistor in the low frequency current path [23,24] or adding a resistor-terminated inductor [25] to absorb the low-frequency single, resulting in improved impedance matching at the low side of the operating band. The last kind is the filtering endfire antenna [26] or patch antenna [27,28] with a resistor-terminated structure. In [26], a resistor-terminated bandstop filter is loaded on the microstrip feed line of the quasi-Yagi antenna to form two radiation nulls due to the two absorption peaks from the loaded structure. In [27,28], a resistor-terminated bandstop filter is loaded on the microstrip feed line of the patch antenna to achieve an out-of-band reflectionless notch response, while the filtering function is achieved by the patch itself.

In this paper, the dual-mode resistor-loaded arms between the driver and the ground and the director closely placed before the driver are utilized to obtain the filtering quasi-Yagi antenna. Different from the resistor-loaded antennas mentioned above, the pair of resistor-loaded arms in the proposed antenna are applied to achieve two additional radiation nulls caused by reverse currents and to absorb the unwanted out-of-band resonant points brought by the arms themselves. The number of radiation nulls can be increased to four, and the compact size along the endfire direction (without ground) can be obtained. The working mechanism of the proposed antenna is analyzed, and a prototype is designed, fabricated, measured, and compared with the state-of-the-art designs.

This paper is organized as follows. In Section 2, the configuration of the proposed filtering quasi-Yagi antenna, the working mechanism of the proposed antenna, and the parametric studies are exhibited. Then, the measured results of the proposed antenna and the comparison with the state-of-the-art designs are provided in Section 3. Finally, the conclusion is given in Section 4.

## 2. Proposed Compact Filtering Quasi-Yagi Antenna with Multiple Radiation Nulls Using Embedded Resistor-Loaded Arms

**Figure 1:**
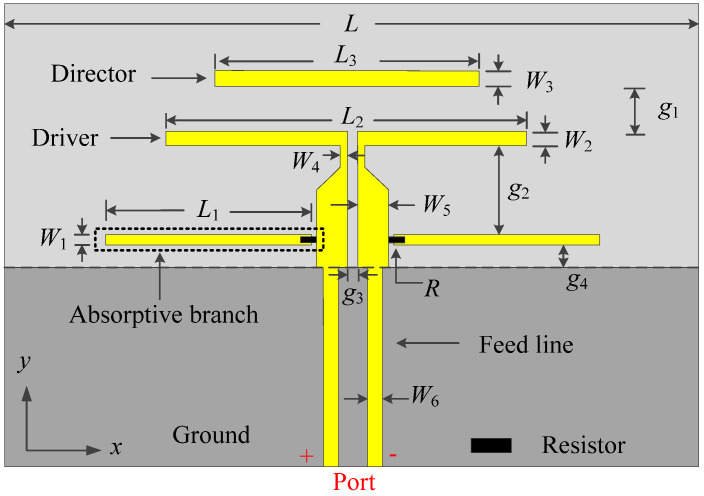
Configuration of the proposed filtering quasi-Yagi antenna.

Figure 1 exhibits the configuration of the proposed filtering quasi-Yagi antenna. The proposed filtering quasi-Yagi antenna consists of a dipole-type driver, a pair of resistor-loaded arms, a director, the ground, and the feed line. The dipole-type driver on the top layer is the main radiator. The pair of resistor-loaded arms is placed close to the ground, between the driver and the ground, in order to provide two additional radiation nulls and absorb the unwanted out-of-band resonant points brought by the arms themselves. Each resistor-loaded arm is composed of a metal strip and a resistor with a resistance of *R*, and the resistor is loaded between the metal strip and the coplanar coupled line. The director, which is positioned closely before the driver on the top layer, provides a resonant point to expand the operating band and a radiation null to increase the upper band-edge frequency selectivity. The ground on the bottom layer works as the reflector. The front end of the coplanar coupled line is chamfered to reduce the effect of the feed line on the driver. The Rogers 4003C substrate with a relative permittivity of 3.38, a loss tangent of 0.0027, and a thickness of 0.508 mm is used in this design. The full-wave simulation is performed by using computer simulation technology (CST).

### 2.1. Antenna Mechanism

**Figure 2:**
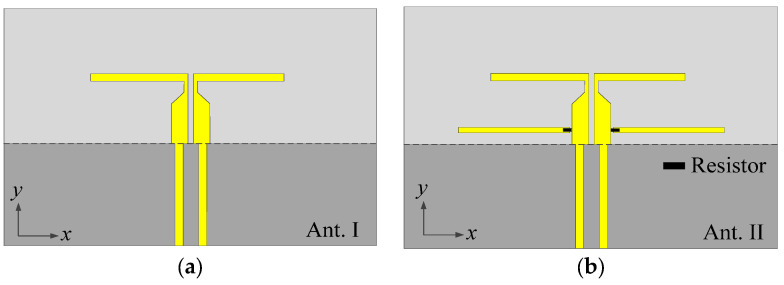
Configurations of the two reference antennas. (**a**) Ant. I. (**b**) Ant. II.

**Figure 3:**
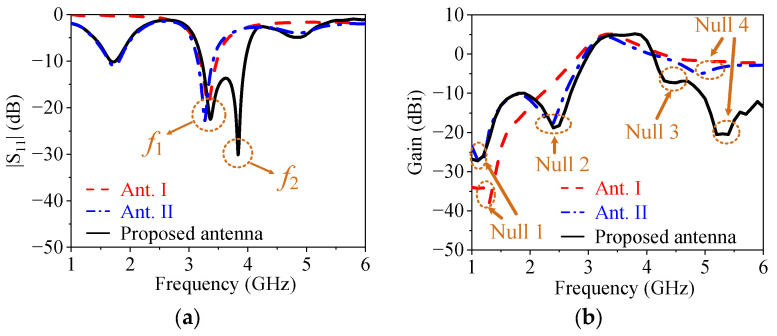
|*S*_11_| and gain of the reference antennas and the proposed antenna. (**a**) |*S*_11_|. (**b**) Gain.

**Figure 4:**
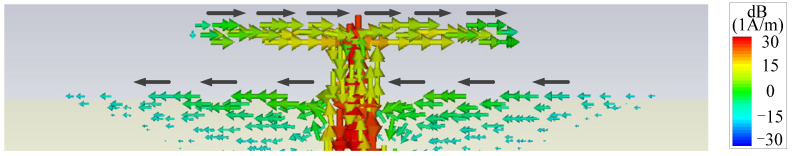
Surface current distribution of Ant. I at Null 1 (The black arrows refer to the currents on the driver and on the front edge of the ground).

In order to understand the working mechanism, the two reference antennas (Ant. I and Ant. II) in Figure 2 are compared with the proposed antenna, and their simulated |*S*_11_| and gain are given in Figure 3. Among them, Ant. I only has one driver except for the ground and the feed line, as shown in Figure 2a. The red curve in Figure 3a shows that Ant. I has a resonant point at *f*_1_ due to the odd mode of the driver. The red curve in Figure 3b exhibits that a radiation null (Null 1) can be achieved at 1.3 GHz. This can be explained by the surface current distribution, as shown in Figure 4, where the current on the driver is opposite to the current on the front edge of the ground, leading to the far-field cancellation in the endfire direction. However, no filtering property can be observed in Ant. I because Null 1 is far away from the operating band.

**Figure 5:**
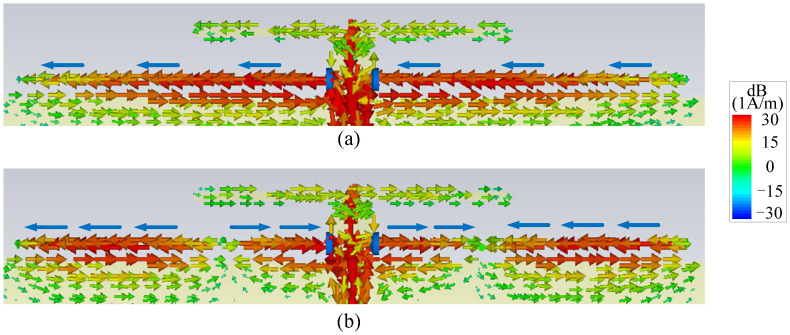
Surface current distributions (**a**) at the lower out-of-band resonant point of Ant. II and (**b**) at the upper out-of-band resonant point of Ant. II (The blue arrows refer to the currents on the resistor-loaded arms).

By embedding a pair of resistor-loaded arms between the driver and the ground, Ant. II can be achieved in Figure 2b on the basis of Ant. I. It can be found from the blue curve in Figure 3a that two more reflection zeros appear outside of the operating band, which correspond to the two resonant points of the resistor-loaded arms. The lower one is the fundamental mode of the resistor-loaded arms, whose electric length corresponds to a quarter wavelength at the lower reflection zero and can be proved by the surface current distribution in Figure 5a. The upper one is the second mode of the resistor-loaded arms, whose electric length corresponds to a three-quarter wavelength at the upper reflection zero and can be proven by the surface current distribution in Figure 5b. Since the arms are close to the ground to form the equivalent transmission line, the open points at the outside ends of the two arms are transformed into the shorted-circuit points at the inside ends of the arms. Thus, the realized gain at these two resonant points is not high because of the absorption from the resistors. The gain reduction in the operating band is very small because the resistors almost do not work when the electric length of each arm is half of a wavelength.

**Figure 6:**
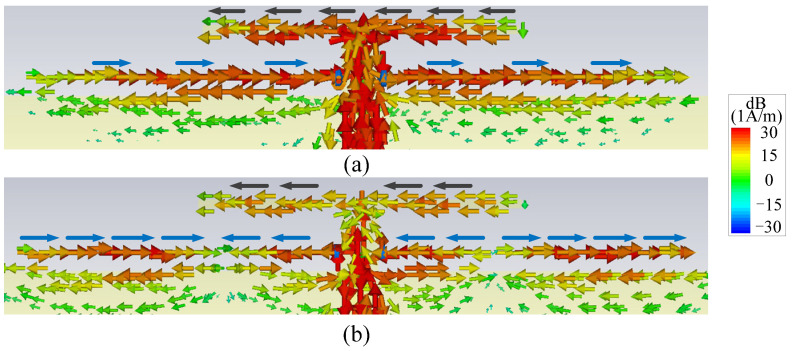
Surface current distributions (**a**) at Null 2 of Ant. II and (**b**) at Null 4 of Ant. II (The black arrows refer to the currents on the driver, and the blue arrows refer to the currents on the resistor-loaded arms).

It is also found from Figure 3b that Null 1 is maintained, and two more radiation nulls (Null 2 and Null 4) can be generated at 2.4 GHz and 4.9 GHz. It is obvious that Null 2 is close to the lower edge of the operating band, so Ant. II has a better frequency selectivity at the lower edge of the operating band when compared with Ant. I. Figure 6 shows the surface current distributions of Ant. II at Null 2 and Null 4 to figure out the reasons for the nulls. It can be seen from Figure 6a that the current distribution on the resistor-loaded arms at Null 2 is close to the fundamental mode. Furthermore, the driver and the resistor-loaded arms have relatively strong currents but with opposite directions, resulting in a far-field cancellation in the endfire direction. At Null 4, the frequency of the radiation null is almost the same as the upper out-of-band resonant point. The resistor-loaded arms exhibit the current of the second mode, and this current is a little stronger than that on the driver. Furthermore, the current at the two sides of the resistor-loaded arms is opposite to the current on the driver and the center part of the resistor-loaded arms, as shown in Figure 6b. Therefore, Null 4 is believed to be caused by both the absorption from the resistor and the far-field cancellation from the reverse currents. Ant. II concludes that although the frequency selectivity at the lower edge of the operating band is improved, the frequency selectivity at the upper edge of the operating band remains poor, and there is just one resonant point in the operating band.

**Figure 7:**
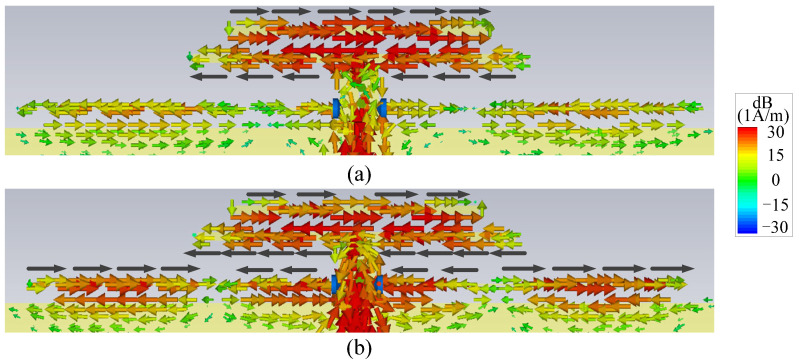
Surface current distributions (**a**) at Null 3 of the proposed antenna and (**b**) at Null 4 of the proposed antenna (The black arrows refer to the currents on the director, the driver and the resistor-loaded arms).

The proposed antenna is formed by adding a director closely before the driver on the basis of Ant. II. It can be seen from Figure 3 that the proposed antenna shows one more resonant point at *f*_2_ to expand the impedance matching bandwidth and one more radiation null (Null 3) to improve the frequency selectivity at the upper edge of the operating band when compared with Ant. II. The second resonant point is caused by the fundamental resonant mode of the director. To find out the reason for Null 3, Figure 7a shows the surface current distributions of the proposed antenna. It can be found that the surface currents mainly distribute on the director and the driver, which are reverse to each other, providing a far-field cancellation in the endfire direction to form Null 3. It can also be found from Figure 3b that Null 4 of the proposed antenna moves upward when compared to the one in Ant. II. This can be explained by the surface current distribution in Figure 7b. Figure 7b shows that the currents on the director and the two sides of the resistor-loaded arms are opposite to the currents on the driver and the center part of the resistor-loaded arms, and the amplitudes of the currents are similar. Thus, it is believed that Null 4 in the proposed antenna is mainly caused by the far-field cancellation from the currents on the resistor-loaded arms, the driver, and the director, leading to the upshift of Null 4.

In summary, the proposed antenna is the preferred one when compared with Ant. I and Ant. II because of more radiation nulls, higher frequency selectivity, wider impedance matching bandwidth, and better out-of-band suppression. 

**Figure 8:**
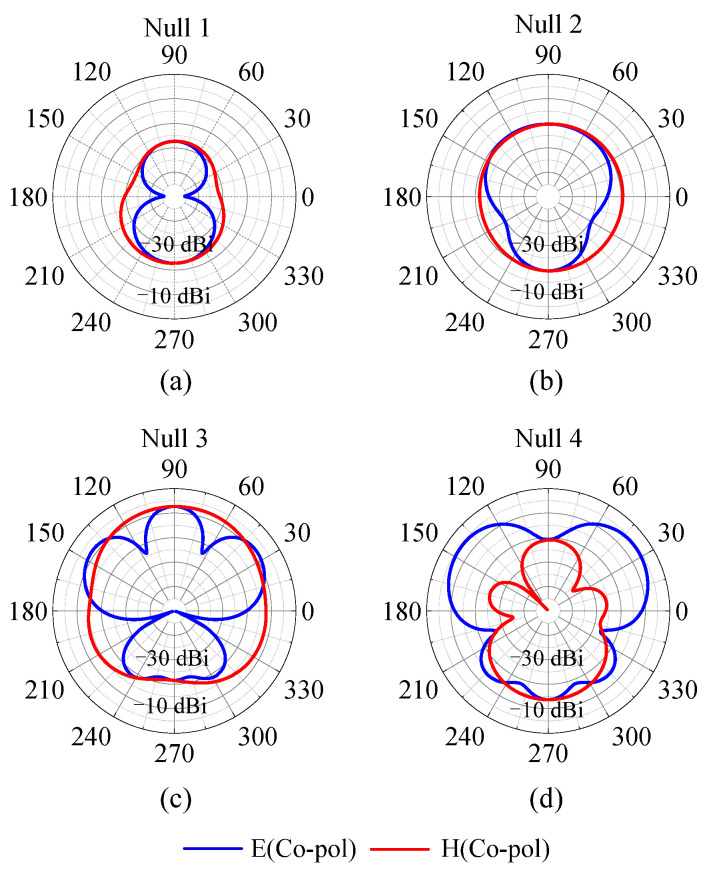
The radiation patterns of the proposed antenna at the frequencies of the radiation nulls. (**a**) The radiation pattern at Null 1. (**b**) The radiation pattern at Null 2. (**c**) The radiation pattern at Null 3. (**d**) The radiation pattern at Null 4.

Figure 8 exhibits the radiation patterns at the four radiation nulls of the proposed antenna. It can be found that the gains in the endfire direction are all very small at the four radiation nulls. The gains in the endfire direction are very close to the maximum gains for Null 1, Null 2, and Null 3. For Null 4, the maximum gain is larger than the gain in the endfire direction because the pair of resistor-loaded arms is larger than 1.5 wavelengths and has opposite currents. 

### 2.2. Parametric Study on L_1_, L_2_, L_3_, g_1_, g_2_, and R

**Figure 9:**
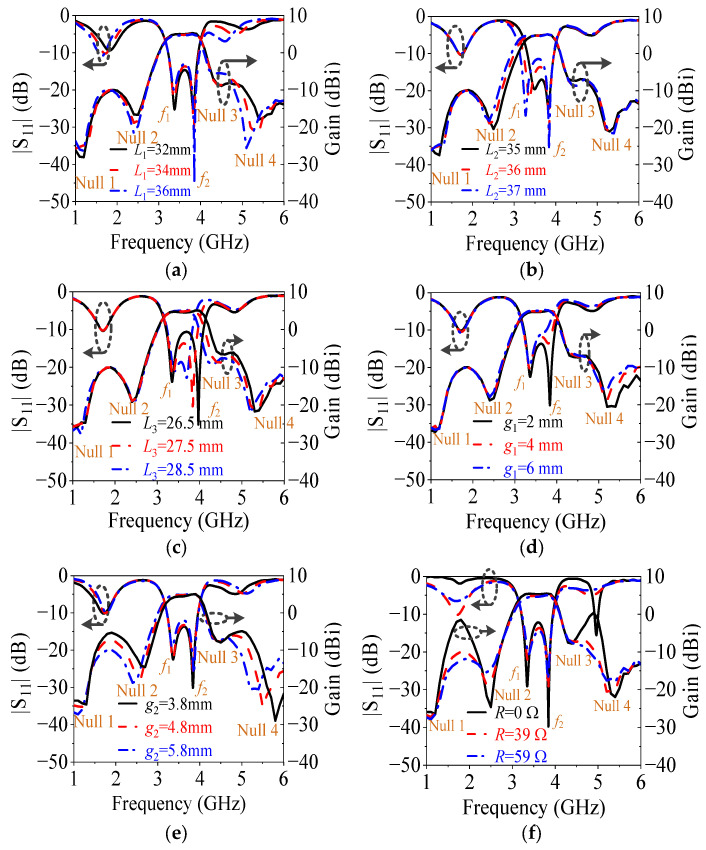
Simulated |*S*_11_| and gain of the proposed filtering quasi-Yagi antenna for different values of (**a**) *L*_1_, (**b**) *L*_2_, (**c**) *L*_3_, (**d**) *g*_1_, (**e**) *g*_2_, and (**f**) *R*.

To illustrate the performance variation, Figure 9 shows the simulated |*S*_11_| and gain of the proposed antenna with different *L*_1_, *L*_2_, *L*_3_, *g*_1_, *g*_2_, and *R*. Figure 9a indicates that the length (*L*_1_) of the resistor-loaded arms mainly affects Null 2, Null 4, and the two reflection zeros outside of the operating band, and they move downward with the increase in the *L*_1_. This is because *L*_1_ determines the mode frequencies of the resistor-loaded arms, which have an important relationship with the formation of Null 2 and Null 4.

Figure 9b exhibits that the length (*L*_2_) of the driver mainly influences *f*_1_ and Null 2. With the increase in the *L*_2_, they move downward. This is because *L*_2_ determines the fundamental odd mode of the driver, and the opposite currents between the driver and the resistor-loaded arms are affected by the driver, as shown in Figure 6a.

Figure 9c shows that the length (*L*_3_) of the director has an impact on *f*_2_, Null 3, and Null 4, and they move upward when *L*_3_ decreases. This is because *L*_3_ determines the fundamental odd mode of the director, and *L*_3_ affects the opposite currents between the director and the driver as shown in Figure 7a, and *L*_3_ also affects the opposite currents between the director, the resistor-loaded arms, and the driver, as shown in Figure 7b.

Figure 9d describes that the gap (*g*_1_) between the director and the driver chiefly has an effect on the bandwidth and Null 4. As *g*_1_ increases, the bandwidth becomes narrow, and Null 4 moves downward because the second resonant frequency and Null 4 are both related to the director.

Figure 9e exhibits that the gap (*g*_2_) between the resistor-loaded arms and the driver mainly affects Null 2, Null 4, the out-of-band suppression level, and the frequency selectivity. With the increase in *g*_2_, the out-of-band suppression level is improved because the equivalent transmission line effect between the arm and the ground increases with *g*_2_, enhancing the open point effect of the outside ends of the arms. Meanwhile, the frequency selectivity is deteriorated, and both Null 2 and Null 4 move downward because the coupling between the driver and the arms is weakened when *g*_2_ increases. Thus, both the out-of-band suppression level and the frequency selectivity need to be considered when choosing the value of *g*_2_. 

Figure 9f shows that the resistance (*R*) of the resistor affects the out-of-band suppression level and the suppression at Null 2 and Null 4. It is found that the out-of-band suppression is very poor if the resistor is not connected (*R* = 0 Ω) because the absorption at these out-of-band resonant points did not happen at this time. As *R* increases, the out-of-band suppression level is improved because the absorption, which is caused by the equivalent short-circuit points at the inside ends of the arms, works. But the suppression at Null 2 and Null 4 is weakened because the current intensity on the arms is reduced to weaken the far-field cancellation. On the other hand, when *R* is equal to 0 Ω, 39 Ω, and 59 Ω, the radiation efficiency of the antenna is 92%, 82%, and 77%, respectively. Therefore, the out-of-band suppression level, the suppression at Null 2 and Null 4, and radiation efficiency should be all considered when choosing the value of *R*.

## 3. Results

**Figure 10:**
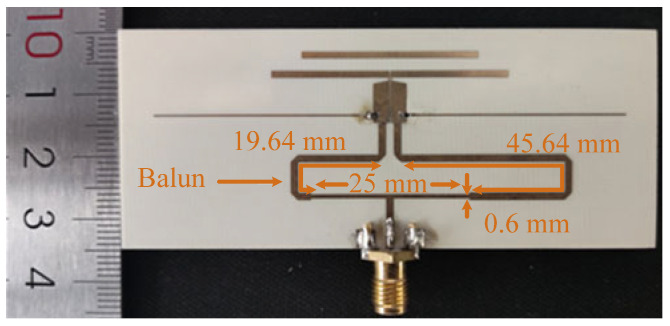
Photograph of the proposed antenna.

Based on the above analysis, a prototype, as shown in Figure 10, is designed and measured. The dimensions of the proposed antenna are shown as follows: *L*_1_ = 34 mm, *W*_1_ = 0.2 mm, *L*_2_ = 36 mm, *W*_2_ = 1 mm, *L*_3_ = 27.5 mm, *W*_3_ = 1 mm, *W*_4_ = 0.1 mm, *W*_5_ = 2 mm, *W*_6_ = 1.1 mm, *g*_1_ = 2 mm, *g*_2_ = 4.8 mm, *g*_3_ = 0.2 mm, *g*_4_ = 2 mm, *L* = 85 mm, and *R* = 39 Ω. The balun is designed for the measurement of the radiation performances, and its dimensions are shown in Figure 10, which is not necessary in a practical differential system. The *S*-parameter of the prototype is measured using the Keysight N5230C vector network analyzer. The gain and the radiation patterns are measured inside an anechoic chamber using a far-field antenna measurement system.

**Figure 11:**
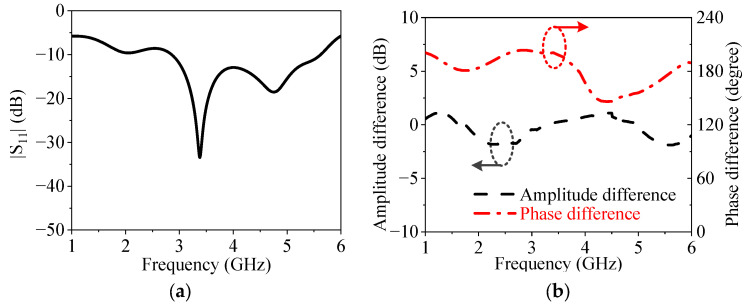
Simulated impedance matching, amplitude difference, and phase difference of the balun. (**a**) |*S*_11_|. (**b**) Amplitude difference and phase difference.

The utilized balun is a traditional microstrip balun using the length difference between the two output paths and has two quarter-wavelength microstrip lines for the impedance matching of the balun. Figure 11 exhibits the simulated impedance matching, amplitude balance, and phase balance of the balun. It can be seen from Figure 11a that the 10 dB impedance matching bandwidth of the balun is from 2.85 GHz to 5.62 GHz, which can cover the operating band of the proposed antenna but cannot cover the lower out-of-band resonant point. From Figure 11b, it can be seen that the amplitude difference between the two output ports of the balun is less than 0.9 dB in the operating band of the antenna, and the phase difference between the two output ports of the balun is around 180° ± 20° in the operating band of the antenna.

**Figure 12:**
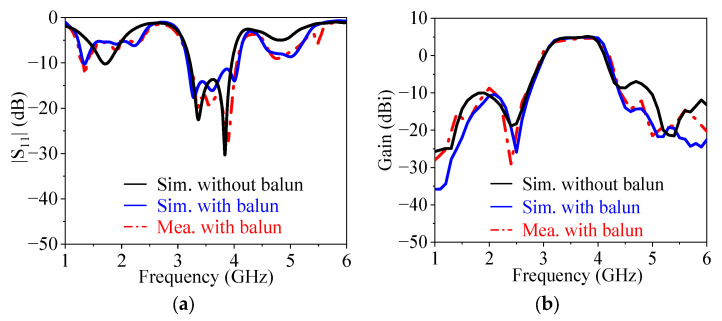
Simulated and measured |*S*_11_| and gain at the endfire direction of the proposed antenna. (**a**) |*S*_11_|. (**b**) Gain at the endfire direction.

Figure 12 exhibits the simulated and measured |*S*_11_| and gain at the endfire direction of the proposed filtering quasi-Yagi antenna. When the balun is introduced, the bandwidth is slightly widened, the out-of-band reflection zeros show ripples, as shown in Figure 12a, and the out-of-band suppression level is improved, as shown in Figure 12b. This is because the bandwidth for the 180° phase difference between the two output ports of the balun is not wide enough to cover the whole frequency range.

**Figure 13:**
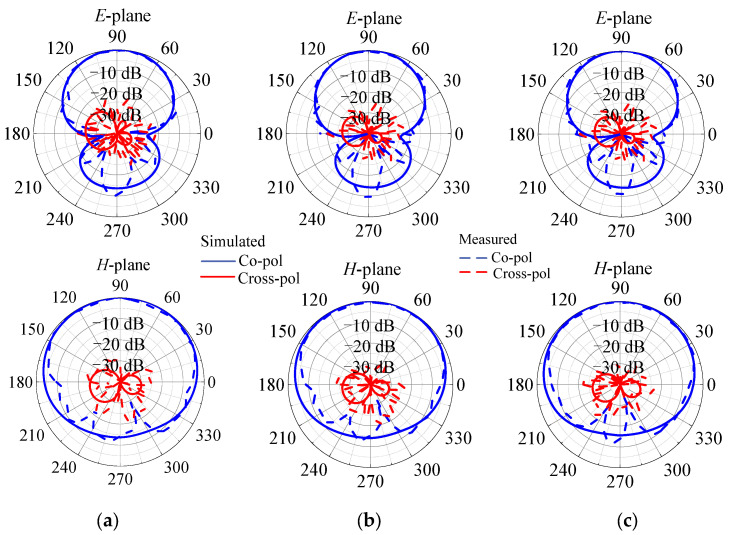
Simulated and measured *E*-plane (*x-y* plane) and *H*-plane (*y-z* plane) radiation patterns of the proposed filtering quasi-Yagi antenna at (**a**) 3.4 GHz, (**b**) 3.6 GHz, and (**c**) 3.8 GHz.

The measured results in Figure 12 indicate that the 10 dB impedance matching bandwidth is from 3.21 GHz to 4.04 GHz, or a fractional bandwidth of 22.9%. The measured peak gain is 4.73 dBi. Four radiation nulls are generated at 1.1 GHz, 2.4 GHz, 4.6 GHz, and 5.2 GHz, respectively. Meanwhile, the out-of-band suppression level is over 13 dB. Figure 13 shows the simulated and measured *E*-plane and *H*-plane radiation patterns at 3.4 GHz, 3.6 GHz, and 3.8 GHz. The measured 3 dB *E*-plane and *H*-plane beamwidths are 66.8°–68.2° and 163.9°–164.7° from 3.4 GHz to 3.8 GHz, respectively. The measured maximum cross-polarization levels in the *E*-plane and *H*-plane within the 3 dB beamwidth are −23.34 and −27.59 dB, respectively.

The performances of the proposed and state-of-the-art filtering quasi-Yagi antennas are listed in Table 1. Compared with the reported filtering quasi-Yagi antennas’ cascading filtering structures, the proposed one can achieve a smaller size along the endfire direction because there is no extra circuit. Compared with the reported filtering quasi-Yagi antennas’ embedding filtering structures, the proposed one can not only achieve the smallest size along the endfire direction, but also obtain the maximum number of radiation nulls. In addition, the bandwidth of the proposed antenna is at a medium level. Therefore, the proposed filtering quasi-Yagi antenna is the preferred one considering the size along the endfire direction, the number of radiation nulls, and the bandwidth.

## 4. Conclusions

In this paper, a compact filtering quasi-Yagi antenna with multiple radiation nulls is proposed. The dual-mode embedded resistor-loaded arms achieve two additional radiation nulls caused by reverse currents and absorb the unwanted out-of-band resonant points brought by themselves. The closely placed director achieves a resonant point and one additional radiation null provided by opposite currents between the driver and the director. Thus, multiple radiation nulls and the compact size along the endfire direction can be achieved, increasing the utility of the antenna. Good endfire radiation can be obtained over a frequency range from 3.21 GHz to 4.04 GHz, which covers the entire 5G band N78. For the purpose of the measurement, a balun is integrated into the filtering quasi-Yagi antenna. A reasonable agreement between the measured and simulated results can be observed.

## Figures and Tables

**Table 1:** The performances of the proposed antenna and the state-of-the-art filtering quasi-Yagi antennas.

Ref. No	Design Method	*f*_0_ (GHz)	FBW (%)	Gain (dBi)	Radiation Nulls	SEFD (Without Ground) (λ_0_)	SEFD (With Ground) (λ_0_)
[4]	Cascading BPF	3.96	14.6	5.82	2	0.55	0.81
[12]	Cascading BSF	4.4	34.1	4.6	5	0.63	0.70
[26]	Cascading resistor-terminated BSF	2.83	11.8	4	2	0.45	0.49
[19]	Embedded DSPSL filter	1.81	2.6	5.6	0	0.36	0.54
[20]	Embedded DSPSL strip	2.3	30.4	5.7	2	0.31	0.35
This work	Embedded resistor-loaded arms	3.62	22.9	4.73	4	0.13	0.33

BPF: bandpass filter; BSF: bandstop filter; FBW: 10 dB fractional bandwidth; SEFD: size along the endfire direction.

## Data Availability

The data presented in this study are available upon request from the corresponding authors.
